# Genome Sequence of the Reuterin-Producing Strain Limosilactobacillus reuteri INIA P572

**DOI:** 10.1128/MRA.00988-21

**Published:** 2021-12-09

**Authors:** Eva Rodríguez-Mínguez, Pol Huedo, Susana Langa, Ángela Peirotén, José M. Landete, Margarita Medina, Juan L. Arqués

**Affiliations:** a Departamento Tecnología de Alimentos, INIA-CSIC, Madrid, Spain; b R&D Department, AB-Biotics S.A. (Part of Kaneka Corporation), Barcelona, Spain; University of Maryland School of Medicine

## Abstract

Limosilactobacillus reuteri is a beneficial bacterium that inhabits the gastrointestinal tract of different mammals. Diverse beneficial effects have been attributed to specific strains, in part mediated by the production of reuterin. Here, we report the draft genome sequence of L. reuteri INIA P572, a reuterin-producing strain isolated from pig feces.

## ANNOUNCEMENT

Limosilactobacillus reuteri INIA P572 was isolated from pig feces collected on a farm near La Coruña (Spain). Stool samples were plated onto Rogosa agar and incubated anaerobically at 37°C for 48 h. Colonies were cultured anaerobically in de Man-Rogosa-Sharpe (MRS) broth at 37°C for 24 h, and the CO_2_-producing isolates were subsequently tested for reuterin production (1). Further analysis identified this strain as reuterin-producing L. reuteri (2) with technological and probiotic properties (2–4), the capacity to grow and produce reuterin in the colonic environment, and *in vivo* immunomodulatory and protective effects (5).

In order to get a deeper knowledge of its safety, as well as the probiotic and technological genomic proprieties of this strain, whole-genome sequencing of L. reuteri INIA P572 was performed. Culturing for genomic DNA (gDNA) isolation, extraction, and library preparation was performed by GenProbio srl (Parma, Italy), according to the method of Lugli et al. (6). Briefly, one isolated colony was grown in MRS broth at 37°C in anaerobic atmosphere. A cell pellet obtained by centrifugation of an overnight culture was used for DNA extraction using the GenElute bacterial genomic DNA kit (Sigma-Aldrich) following the manufacturer’s guidelines. Quantification of DNA was performed using NanoDrop. A genome library was generated using the TruSeq Nano DNA kit. The genome sequences were retrieved using an Illumina MiSeq platform with 2 × 250-bp paired-end reads. Sequencing generated 539,226 reads that were quality controlled using FastQC and assembled using MIRA v4.0.2 software (7), and evaluation of the final assembly was conducted using QUAST v4.3 (8) and CheckM v1.0.7 (9) (with the options lineage_wf and reduced_tree). The genome was annotated using the NCBI Prokaryotic Genome Annotation Pipeline (PGAP) v4.13 (10). The presence of antimicrobial resistance genes was inspected in July 2021 using the last version of the Comprehensive Antimicrobial Resistance Database (CARD) (11) and verified using the ResFinder v4.00 tool (12), with default parameters in both cases.

Genome sequencing and assembly of L. reuteri INIA P572 resulted in 180 contigs, comprising 2,113,987 bp with an *N*_50_ value of 27,758 bp, a 113.83× coverage, and a G+C content of 39.0%. PGAP annotation predicted 2,200 genes, including 1,948 protein-coding sequences. It also identified 73 tRNA sequences, 6 5S rRNA sequences, 13 16S rRNA sequences, and 10 23S rRNA sequences.

The susceptibility of L. reuteri INIA P572 to a panel of antibiotics was determined by the MICs following the standard ISO 10932:2010 IDF 223:210 (2010) and EFSA guidelines (13). L. reuteri INIA P572 was phenotypically resistant to ampicillin, chloramphenicol, and tetracycline (Table 1). CARD and ResFinder analyses identified the tetracycline resistance gene *tetW* in its genome. The presence of this gene hampers the use of this strain as food supplement, according to EFSA requirements (13, 14).

**TABLE 1:** MIC values for L. reuteri INIA P572 of the antibiotics included in the EFSA guidelines^*a*^

Antibiotic	MIC (mg/liter)	EFSA cutoff (mg/liter)	Outcome
Ampicillin	4	2	R
Vancomycin	n.r.	n.r.	n.r.
Gentamicin	2	8	S
Kanamycin	64	64	S
Streptomycin	16	64	S
Erythromycin	0.5	1	S
Clindamycin	1	4	S
Tetracycline	>64	32	R
Chloramphenicol	8	4	R

a n.r., not required; R, resistant; S, susceptible.

### Data availability.

The whole-genome sequencing project for L. reuteri INIA P572 has been deposited at GenBank under the accession number NZ_CABFNG000000000.1. The raw sequencing data are available in the Sequence Read Archive (SRA) database under accession number SRR16079620. The associated BioProject accession number is PRJNA766497.
